# Profiling arbutin as a potent cholinesterase and GSK-3β inhibitor and evaluating its cytotoxicity in SH-SY5Y cells for Alzheimer’s disease therapy

**DOI:** 10.55730/1300-0152.2972

**Published:** 2026-06-01

**Authors:** Shirin TARBIAT, Tuğba BAL, Deniz GÜLMEZ

**Affiliations:** 1 Department of Molecular Biology and Genetics, Faculty of Engineering and Natural Sciences, Üsküdar University, İstanbul, Turkiye; 2 Graduate School of Sciences, Üsküdar University, İstanbul, Turkiye; 3 Department of Bioengineering, Faculty of Engineering and Natural Sciences, Üsküdar University, İstanbul, Turkiye

**Keywords:** Arbutin, AChE, BuChE, GSK-3β, Alzheimer’s disease

## Abstract

**Background/aim:**

Alzheimer’s disease (AD) is a neurodegenerative disorder linked to cognitive decline and memory loss, marked by hyperphosphorylated tau tangles and decreased acetylcholine levels, which are affected by acetylcholinesterase (AChE) and butyrylcholinesterase (BuChE) activity. Glycogen synthase kinase-3 beta (GSK-3β) also plays a role in AD through abnormal activation. In this study, the potential of β-arbutin to inhibit cholinesterases and GSK-3β was investigated, utilizing in vitro assays, cytotoxicity analysis, and molecular docking simulations to explore its therapeutic implications.

**Materials and methods:**

Ellman’s technique was used to test the inhibitory effects of arbutin on cholinesterase enzymes. An ATP-Glo luminescent assay kit was used to evaluate the inhibitory effects and inhibition kinetics of arbutin on GSK-3β. A Cell Titer-Glo 2.0 assay kit was used to assess the cytotoxicity of arbutin in SH-SY5Y cells.

**Results:**

β-Arbutin exhibited significant inhibitory effects on AChE, BuChE, and GSK-3β, with IC_50_ values of 0.6, 4, and 1.23 μM, respectively. The inhibition kinetics of GSK-3β with respect to ATP was competitive but with respect to the substrate (GS-2) was noncompetitive. We further found that β-arbutin, when applied alone to SH-SY5Y cells, does not affect cell viability and can ensure the sustainability of cell viability and health. Docking scores were −7.980, −6.726, and −6.115 kcal/mol for GSK-3β, AChE, and BuChE, respectively.

**Conclusion:**

Our research demonstrates that arbutin is a modulator of cholinesterase and GSK-3β and may be beneficial as a multitarget-directed therapeutic natural compound for AD.

## 1. Introduction

Alzheimer’s disease (AD) is a chronic and progressive neurodegenerative disorder characterized by cognitive decline and memory impairment. Disease is the most common cause of dementia, accounting for 60–80% of dementia cases. Because of the global increase in life expectancy, high mortality rates, and lack of effective therapeutic agents for treatment and prevention, there is an urgent need for effective therapeutic strategies. Current research has revealed that AD neuropathology is triggered by the abnormal accumulation of amyloid-β (Aβ) plaques, increased inflammation, hyperphosphorylated tau neurofibrillary tangles, reduced acetylcholine (ACh) levels, and mitochondrial impairment (Pinky et al., 2026). Given the limited number of approved drugs and their inability to halt disease progression, the multifaceted nature of this complex disorder is evident, underscoring the importance of identifying novel molecular targets and bioactive reagents with potential therapeutic relevance (Li et al., 2025).

Research has found key molecular processes linked to AD. The cholinergic hypothesis is a main theory explaining memory loss, suggesting that low levels of ACh in neurons cause cognitive problems. ACh is crucial for learning and memory, and its quick breakdown is caused by cholinesterase enzymes, namely acetylcholinesterase (AChE) and butyrylcholinesterase (BuChE). AChE is vital for acetylcholine hydrolysis in cholinergic synapses, terminating nerve impulses. Increased AChE activity diminishes ACh levels and accelerates Aβ fibril clumping. As AD progresses, AChE activity declines, and BuChE becomes more significant in ACh hydrolysis, with BuChE overexpression converting nonfibrillar to fibrillary Aβ, leading to senile plaques. Hence, inhibiting both cholinesterases is a key therapeutic approach to maintain ACh levels and enhance cognitive function in Alzheimer’s patients. Aberrant intracellular signaling pathways, particularly the overactivation of glycogen synthase kinase-3 beta (GSK-3β), significantly contribute to AD pathology. GSK-3β is linked to critical processes such as tau hyperphosphorylation, synaptic dysfunction, neuroinflammation, and neuronal apoptosis. Its dysregulated activity fosters neurofibrillary tangles from abnormally phosphorylated tau, accelerating AD progression. Consequently, targeting both cholinesterase activity and GSK-3β may yield comprehensive therapeutic benefits for AD (Almaghrabi, 2025).

**Figure 1:**
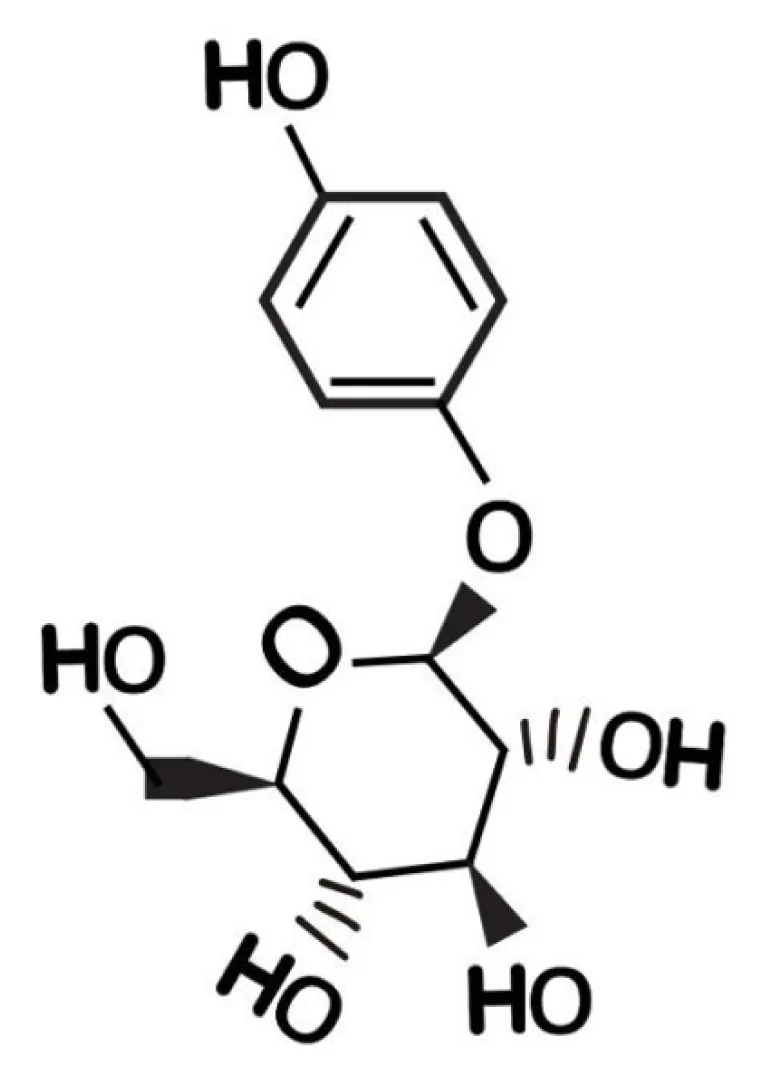
Chemical structure of arbutin (illustrated by the authors).

Arbutin (p-hydroxyphenyl-β-D-glucopyranoside) as presented in Figure 1 is a bioactive hydrophilic polyphenol. Arbutin has two isomers, namely α-arbutin (4-hydroxyphenyl-α-glucopyranoside) and β-arbutin (4-hydroxyphenyl-β-glucopyranoside). The formation of isomers of arbutin is determined by the binding of hydroquinone to the anomeric carbon of glucose. β-Arbutin or arbutin is naturally occurring, while α-arbutin is synthesized enzymatically (Saeedi et al., 2021). Major sources of β-arbutin include the families Asteraceae, Ericaceae, Proteaceae, and Rosaceae, and it is valued in cosmetics for its antioxidant and skin depigmentation properties (Migas and Krauze-Baranowska, 2015). Its antimicrobial, antiinflammatory, and anticancer effects have also been investigated. It has shown potential in reducing risks for neurodegenerative diseases, yet limited studies address its impact on AD (Nahar et al., 2022; Temgire et al., 2025). In the present study, the effects of β-arbutin on AChE, BuChE, and GSK-3β were comprehensively investigated through integrated in vitro and in silico approaches. To the best of our knowledge, this is the first study on the inhibitory activity and IC_50_ values of β-arbutin against AChE, BuChE, and GSK-3β, as well as being the first study providing kinetic characterization of GSK-3β inhibition by β-arbutin. In addition, molecular docking analysis and cytotoxicity evaluation in SH-SY5Y neuroblastoma cells were performed to further characterize its pharmacological profile. These findings provide novel insights into the multitarget potential of β-arbutin and highlight its relevance for further in vivo and clinical investigations in AD management.

## 2. Materials and methods

### 2.1. Cholinesterase assay

Ellman’s technique was used to test the inhibitory effects of arbutin on AChE and BuChE. Arbutin (catalog number 66468-50MG) and the other chemicals used for these assays were acquired from Sigma-Aldrich Co. (St. Louis, MO, USA). The arbutin’s inhibitory activity was measured using a mixture that included 200 μL of an AChE/BuChE solution (0.415 U/mL in 0.1 M phosphate buffer, pH 8.0), 100 μL of a 5,5′-dithiobis-(2-nitrobenzoic acid) (DTNB) solution (3.3 mM in 0.1 M phosphate-buffered solution, pH 7.0) containing NaHCO_3_ (6 mM), and 500 μL of phosphate buffer, 0.1 M, pH 8.0. The substrate, 100 μL of a 0.1 mM acetylcholine iodide/butyryl thiocholine chloride solution, was applied to the cuvette following 20 min of incubation at 25 °C. The AChE/BuChE activity was determined by measuring the change in absorbance at 412 nm for 3 min at 25 °C using a spectrophotometer (UV-2600, Shimadzu, Kyoto, Japan). Initially, for the inhibition study, an aqueous solution of arbutin (10–200 μg/mL) was incubated for 5 min with the enzymes. The brains of recently sacrificed, healthy adult Wistar rats were used to isolate the enzymes AChE and BuChE. Using a glass Teflon homogenizer, brain tissues were homogenized in ice-cold 0.1 M phosphate buffer (pH 7.4). They were then centrifuged at 10,000 × *g* for 15 min at 4 °C. After cell lysates were collected, the supernatant was used as the source of crude enzymes for the inhibition tests. Galantamine hydrobromide was used as a reference inhibitor for AChE and BuChE in concentration ranges of 0.05–3 μg/mL and 0.3–30 μg/mL, respectively (Dirie et al., 2025). Crude enzyme preparations were used in the present study to preserve the native biochemical environment of cholinesterases and to allow preliminary evaluation of inhibitory activity under physiologically relevant conditions (Girgin et al., 2024). All experiments were performed in triplicate under standardized experimental conditions to minimize the variability associated with tissue-derived crude enzyme preparations.

### 2.2. GSK-3β assay

The GSK-3β Kinase Enzyme System kit (catalog number: V1991) was purchased from Promega GmbH (Walldorf, Germany). The assay was performed according to previous studies, by adding 1 μL of active GSK-3β enzyme solution (0.1 μg/μL) to each well, followed by 2 μL of 1x assay buffer (prepared by diluting 5x supplemented buffer containing 50 μM DTT and 50 μM ATP). Then 2 μL of substrate (GS-2, glycogen synthase-derived peptide) solution (0.3 μg/μL) was added, bringing the total reaction volume to 5 μL. For the inhibition study, a 500-μM stock solution of arbutin was prepared in double-distilled water and subsequently diluted with the assay working buffer to obtain final concentrations ranging from 3 to 25 μM. A volume of 1 μL of each test concentration of arbutin was added to the wells of a 384-well low-volume plate and preincubated with 1 μL of enzyme solution, followed by addition of the reaction mixture as in the enzyme assay.

The reaction mixture in all wells, with the absence (control) and presence of arbutin, was incubated at 25 °C for 60 min. Later, 5 μL of ADP-Glo reagent was added, followed by incubation for an additional 40 min at 25 °C. Subsequently, 10 μL of kinase detection reagent was added and the plate was incubated for a further 30 min at 25 °C. Luminescence was then measured to determine the inhibitory activity of arbutin against GSK-3β (Liu et al., 2022).

### 2.3. Mechanism of inhibition on GSK-3β: kinetic studies

#### 2.3.1. Jump dilution assay

To verify the reversibility of enzyme inhibition, a jump dilution assay was conducted prior to kinetic analysis to reduce inhibitor concentration below its IC_50_ value. The procedure involved preincubating the enzyme with a high inhibitor concentration (125 μM, 10× IC_50_) or without an inhibitor, followed by rapid dilution with a reaction buffer to initiate phosphorylation. This dilution lowered the inhibitor concentration to approximately 0.125 μM while preserving enzyme activity. Reversible inhibition was evidenced by the restoration of enzyme activity postdilution, contrasting with prolonged activity reduction indicative of irreversible inhibition. Residual enzyme activity was measured relative to the control, expressed as mean ± SD from two independent experiments (Liu et al., 2021).

#### 2.3.2. Determination of inhibition type

The kinetic experiments were performed to investigate the inhibitory mechanism of arbutin on GSK-3β. Lineweaver–Burk plots of enzyme kinetics with varying inhibitor concentrations of 0.5–3 μM were performed. GSK-3β is a bisubstrate kinase enzyme that utilizes ATP and GS-2 peptide as substrates. To determine the inhibition mechanism, initially, the concentration of ATP was set at 10–50 μM, while the concentration of substrate (GS-2) was kept constant at 0.2 (μg/μL). Then the substrate concentration was set at 0.05–0.3 μg/μL, while the ATP concentration remained unchanged (Liu et al., 2021). Enzyme kinetic data were analyzed using the software GraphPad Prism (GraphPad Software, Boston, MA, USA) using nonlinear regression and enzyme kinetic evaluation in combination with Lineweaver–Burk transformation plots to support interpretation of the inhibition mechanism.

### 2.4. Cytotoxicity assay

The cytotoxicity of arbutin on SH-SY5Y cells was assessed using the Cell Titer-Glo 2.0 assay. SH-SY5Y cells were cultured in 96-well plates at a density of 10^4^ cells per well for 24 h at 37 °C in 5% CO_2_ for adherence. They were then treated with arbutin at concentrations between 50 and 1000 μM, alongside a control group, and incubated for another 24 h. Following this, the culture medium was removed and 50% Cell Titer-Glo 2.0 reagent (prepared in Dulbecco’s modified Eagle’s medium (DMEM)) was added to each well. The mixture was shaken in the dark for 2 min at 59 rpm and then incubated at room temperature for 10 min to stabilize the luminescent signal. Following this, 200 μL from each well was transferred to an opaque plate for luminescence measurement using a BMG LABTECH plate reader. A blank control with DMEM and Cell Titer-Glo 2.0 reagent was used to account for background luminescence (Bal et al., 2019).

### 2.5. Protein and ligand preparation

The crystal structures of the target proteins, GSK-3β, AChE, and BuChE, were retrieved from the RCSB Protein Data Bank, with PDB IDs 1J1C, 4EY6, and 6AQQ, respectively. Molecular docking was conducted using Schrödinger Maestro (2025-1). The preparation steps included filling missing side chains, optimizing H-bond assignments with PROPKA, and minimization through the OPLS4 force field (Chavan et al., 2025; Sarnow et al., 2025). Only the target protein chain, waters within 5 Å of the ligand, and the crystal ligand were retained, with the Mg^2+^ ion of GSK-3β preserved. The default position was used for residues with alternative positions. For docking, arbutin’s 2D structure (CID: 440936) was procured from PubChem and prepared with LigPrep, generating possible states at pH 7.0 ± 2.0 with Epik Classic (Choutka et al., 2025).

### 2.6. Molecular docking

The protein grid boxes were centered around specific ligand binding sites: the ADP site for GSK-2β, the (−)-galantamine site for AChE, and the (S)-2-(butylamino)-N-(2-cycloheptylethyl)-3-(1H-indol-3-yl) propenamide site for BuChE. The ligands were allowed to be flexible, with Epik state penalties included in the docking score. Docking utilized the Glide SP module of Schrödinger Maestro (2025-1) (Kantarci-Carsibasi et al., 2025), validated by docking native ligands in the crystal structures and calculating Glide RMSD values of 0.790 Å for GSK-3β, 0.160 Å for AChE, and 0.639 Å for BuChE.

### 2.7. Statistical analysis

All data from the experiments were collected in triplicate and analyzed using GraphPad Prism version 9.5.1 (GraphPad Software, San Diego, CA, USA). The results from three separate experiments were expressed as mean ± standard deviation.

## 3. Results

### 3.1. Cholinesterase inhibition

An in vitro enzyme inhibition test was conducted to evaluate arbutin’s ability to inhibit cholinesterases, with galantamine serving as a positive control. The results are shown in Table. Arbutin demonstrated concentration-dependent inhibition of AChE activity, with an IC_50_ value of 0.6 ± 0.4 μM. Galantamine, under the same conditions, exhibited an IC_50_ value of 0.69 ± 0.2 μM for AChE inhibition. Arbutin also inhibited BuChE in a concentration-dependent manner, resulting in an IC_50_ value of 4 ± 0.4 μM. Galantamine under the same experimental conditions inhibited BuChE with an IC_50_ value of 2.83 ± 0.3 μM.

### 3.2. GSK-3β inhibition

The in vitro inhibitory activity of arbutin against GSK-3β was evaluated using the Kinase-Glo luminescent kinase assay, with staurosporine used as the reference inhibitor. The results are presented in Table. Dose–response analyses demonstrated that arbutin inhibited GSK-3β activity in a concentration-dependent manner, yielding an IC_50_ value of 1.25 ± 0.03 μM. Under identical experimental conditions, staurosporine exhibited substantially stronger inhibitory activity, with an IC_50_ value of 0.036 ± 0.001 μM.

### 3.3. Kinetic parameters of GSK-3β inhibition

#### 3.3.1. Jump dilution assay

**Figure 2:**
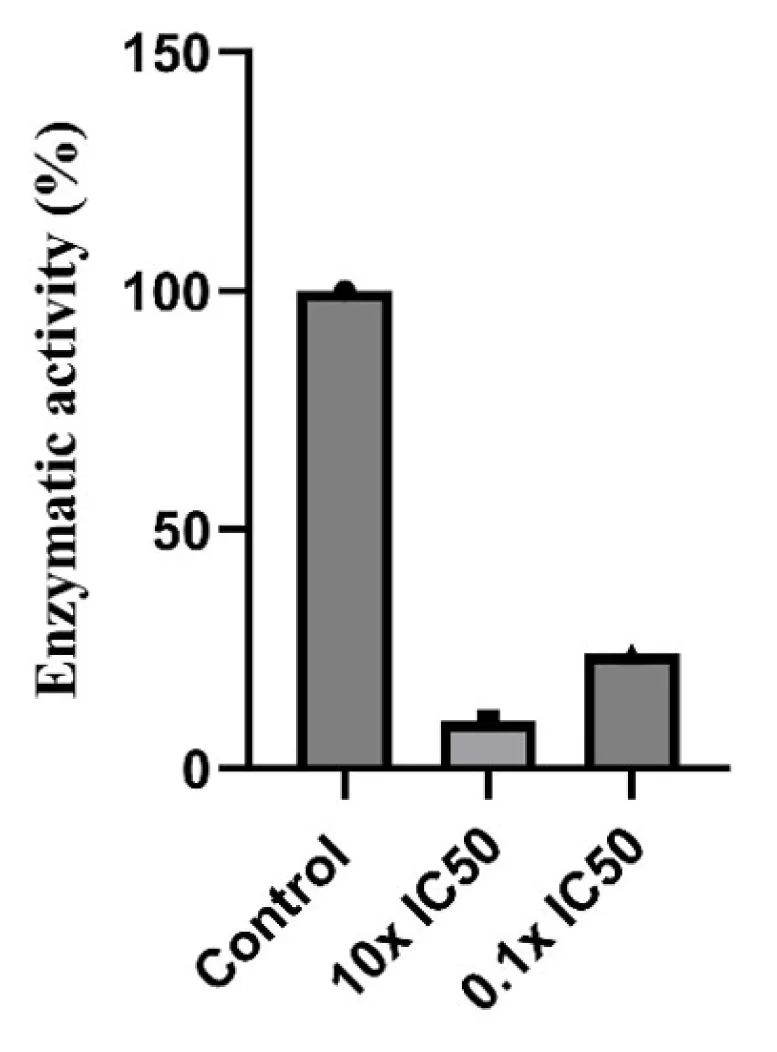
Jump dilution experiment indicating enzymatic activity in preincubation with arbutin (10× IC_50_) and after dilution (0.1× IC_50_).

A jump dilution test was used to evaluate the reversibility of enzyme inhibition. Enzymatic activity was assessed under control conditions, preincubated with arbutin at 10× IC_50_, and by rapid dilution to its 0.1× IC_50_ conditions. Enzyme activity in the control group was adjusted to 100%, as seen in Figure 2. Enzymatic activity was significantly lower after preincubation with the inhibitor at 10× IC_50_, resulting in a marked reduction in enzymatic activity, with residual activity remaining below 15%. Upon dilution to 0.1× IC_50_, only limited recovery of activity was observed; upon dilution to 10× IC_50_, about 20–30% of control values were seen. The partial recovery of enzyme activity after dilution suggests that the test conditions indicated partial or relatively slow recovery of enzyme activity after dilution.

#### 3.3.2. Inhibition kinetics of GSK-3β with respect to ATP

**Figure 3:**
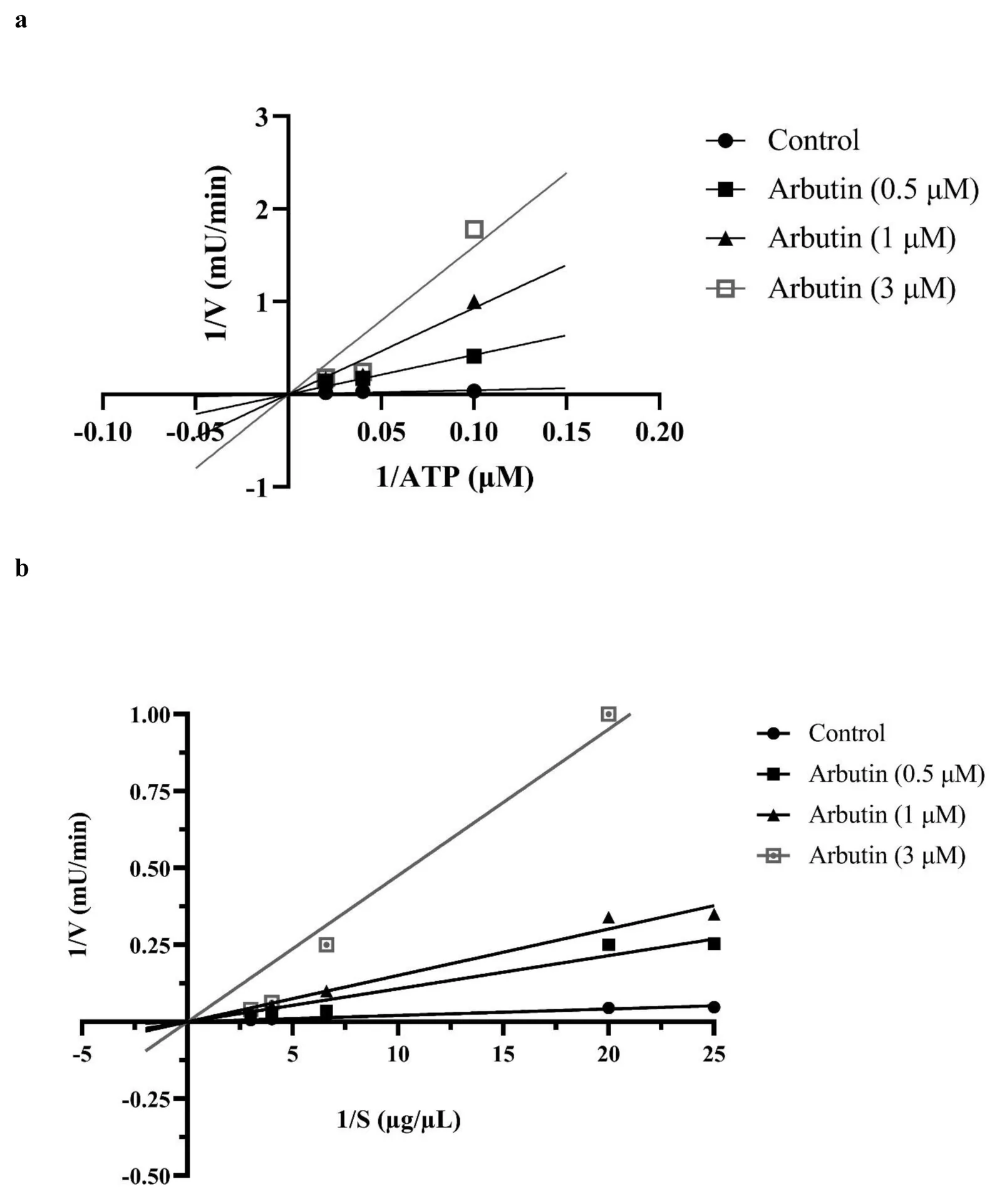
Lineweaver–Burk plots illustrating the effect of increasing arbutin concentrations on GSK-3β activity by varying substrate concentration to determine the type of inhibition. (a) Competitive inhibition with respect to ATP. All lines meet at the same y-intercept with increased slopes. (b) Noncompetitive inhibition with respect to GS-2; the lines cross at the same x-intercept. The y-intercept increases as arbutin concentration increases.

Enzyme kinetic analysis was performed using Lineweaver–Burk plots with ATP as the substrate at increasing concentrations of arbutin, as shown in Figure 3a. The resulting plots show the same y-intercept (1/V_max_) with progressively increasing slopes as inhibitor concentration increases, while the x-intercept shifts toward zero. V_max_ remains unchanged while K_m_ increases with arbutin concentration. This kinetic pattern suggests that the inhibitor and ATP compete for binding to the active site, a characteristic of competitive inhibition with respect to ATP. Nonlinear regression analysis using GraphPad Prism revealed an inhibition constant (K_i_) of 0.03962 μM, demonstrating a high affinity of arbutin for the enzyme.

#### 3.3.3. Inhibition kinetics of GSK-3β with respect to substrate (GS-2)

Enzyme kinetic analysis of GSK-3β was performed using Lineweaver–Burk plots at varying concentrations of arbutin and GS-2, as shown in Figure 3b. All lines intersect at (or very close to) the same x-intercept. The y-intercept increases as arbutin concentration increases. K_m_ remains unchanged while V_max_ decreases. This indicates that increasing substrate concentration does not overcome inhibition. The arbutin binds to an allosteric site (not the active site). Substrate binding remains unaffected; thus, K_m_ is unchanged. Catalytic efficiency is reduced, resulting in decreased V_max_. The inhibition constant (K_i_) determined by nonlinear regression analysis was 0.2462 (μg/μL). Different K_i_ units were used depending on the substrate applied in the GSK-3β kinetics experiments. ATP-based analyses were expressed in μM because ATP’s molecular weight is known, whereas GS-2-based analyses were expressed in μg/μL according to the commercial kit specifications, since the molecular weight of GS-2 was not provided.

### 3.4. Cytotoxicity assessment on SH-SY5Y cell lines

**Figure 4:**
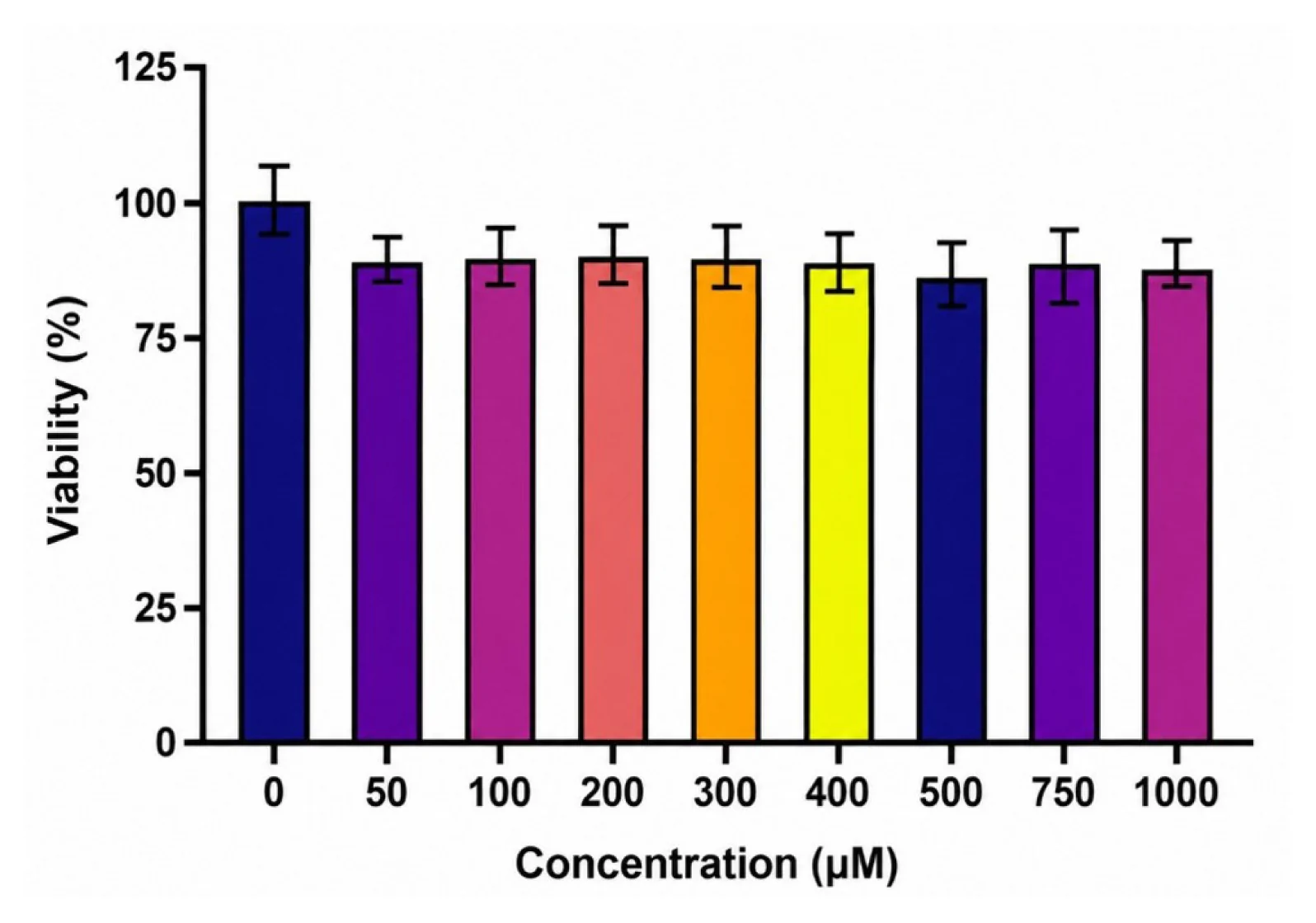
Dose-dependent effect of arbutin on SH-SY5Y cell viability. The difference between the control and experimental groups was not statistically significant.

In a study with SH-SY5Y cells, arbutin was assessed for its effect on cell viability at various concentrations, as shown in Figure 4. The results showed that cell viability remained high (85–90%) across all groups treated, with the lowest viability at 500 μM (85.59%) and the highest at 200 μM (89.40%). No significant decrease in cell viability was found related to concentration, indicating consistent tolerance of cells to arbutin across different doses and the reliability of the experimental data.

### 3.5. Molecular docking

**Figure 5:**
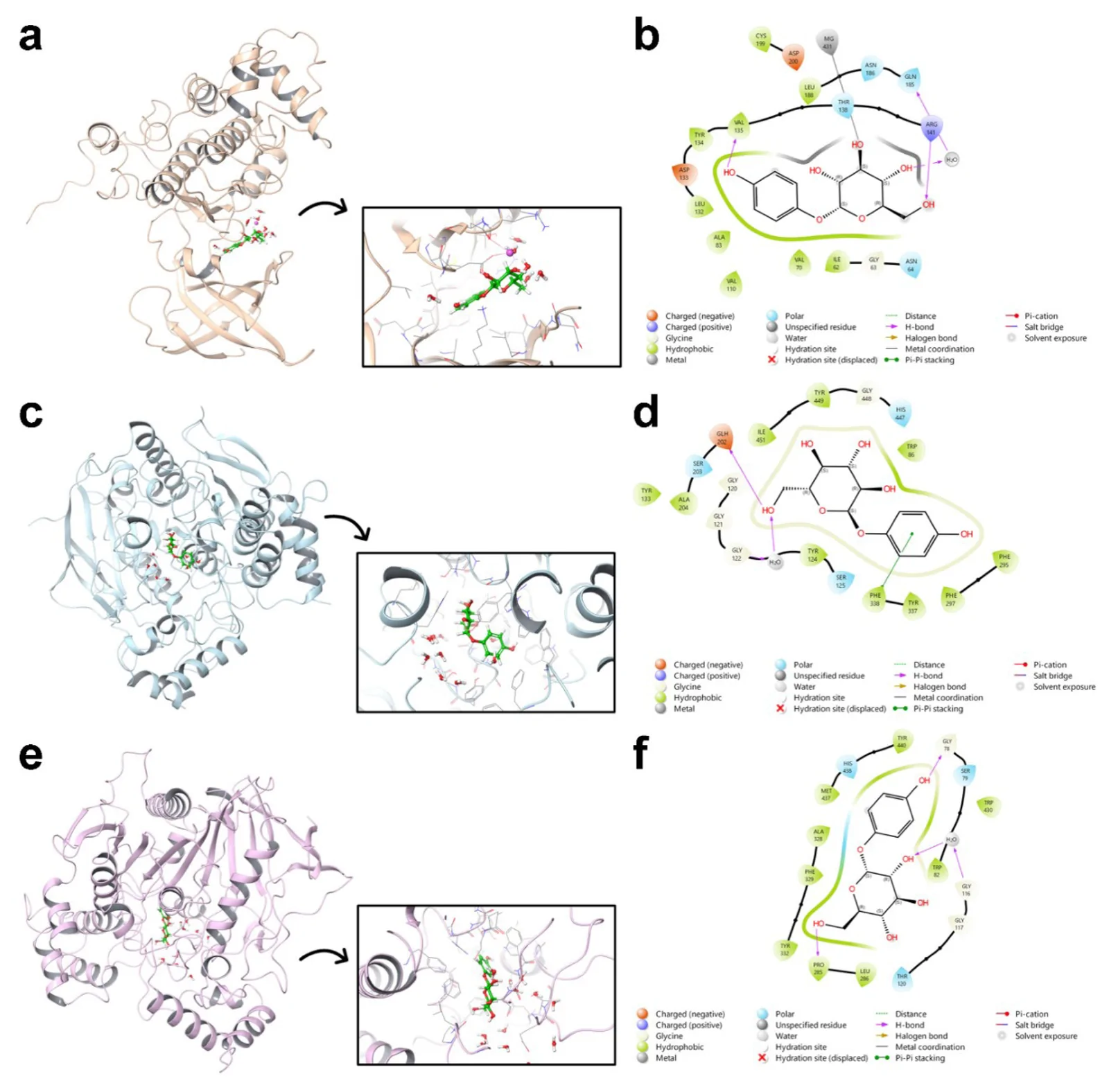
Molecular docking of arbutin to GSK-3β (a, b), AChE (c, d), and BuChE (e, f). (a, c, e) Arbutin in the docked pose within the protein structure. (b, d, f) 2D ligand–protein interaction maps.

Molecular docking studies showed moderate binding of the arbutin to the target regions within each protein (Figure 5). Docking scores of −7.980, −6.726, and −6.115 kcal/mol were obtained for GSK-3β, AChE, and BuChE, respectively, and the docked pose of arbutin within each binding region is illustrated in Figures 5a, 5c, and 5e. In docking with GSK-3β, further investigation of arbutin localization revealed that a hydroxyl group of D-glucose of arbutin undergoes metal coordination with Mg^2+^, and another hydroxyl group of the D-glucose moiety interacts with ARG141. Additionally, the hydroquinone moiety of arbutin establishes a hydrogen bond with VAL135, as shown in Figure 5b. In contrast, in docking analysis with AChE, GLH202 is hydrogen-bonded with arbutin and PHE338 forms pi–pi stacking with the hydroquinone of the arbutin, as shown in Figure 5d. Finally, BuChE interactions showed that PRO285, which was reported as one of the amino acids interacting in BuChE inhibitor studies, and the other amino acid, GLY78, form hydrogen bonds with arbutin, as shown in Figure 5f. For AChE and BChE docking, water-mediated bindings were also observed.

## 4. Discussion

AChE inhibition continues to be a major approach in the symptomatic treatment of AD, because decreased synaptic ACh levels are strongly related to the cognitive deficit (Neha and Parvez, 2023). In the present study, the active compound, arbutin, showed potent AChE inhibition, with an IC_50_ value slightly lower than that of the positive control, galantamine, a clinically established AChE inhibitor. Arbutin’s inhibitory potency, when compared to the reference inhibitor, suggests effective AChE inhibition and supports its potential to influence the cholinergic pathway. The observed potency of arbutin indicates that it may efficiently interact with the active site of AChE, demonstrating similar efficacy and inhibitory mechanisms as the positive control. The inhibitory potency of arbutin in the submicromolar range suggests a pharmacologically relevant affinity toward AChE, consistent with the importance of IC_50_ values as key indicators of inhibitor potency in early-stage drug discovery (Srinivasan, 2023). Previous studies have shown that compounds exhibiting low to submicromolar IC_50_ values are generally considered promising candidates for further pharmacological investigation (Žužek, 2024).

BuChE has become of growing interest as a complementary therapeutic target in AD, particularly in later disease stages when AChE activity declines and BuChE acts dominantly (Sang et al., 2025). In the current investigation, arbutin was found to have a moderate level of inhibitory action on BuChE compared to the positive control. Even though the active compound demonstrated reduced efficacy against BuChE relative to the reference inhibitor, it maintained significant inhibitory activity in the low micromolar range. This inhibitory potency remains significant when compared to highly active small polyphenolic derivatives, with IC_50_ values ranging between 2 and 20 μM (Wu et al., 2022).

The lower level of BuChE inhibition compared to that of AChE may imply a certain degree of selectivity towards AChE, which could be beneficial to elide peripheral cholinergic side-effects related to strong BuChE inhibition (Zhou and Huang, 2022).

Hyperphosphorylated tau protein is crucial for microtubule formation in neurons. In AD, tau hyperphosphorylation hinders microtubule assembly, leading to neuronal damage (Jiang et al., 2025).

Despite the promising inhibitory activities observed in the present study, certain methodological limitations should be considered when interpreting the findings. Although crude enzyme preparations provide a biologically relevant environment, they may contain endogenous biomolecules that could influence enzymatic activity and contribute to variability between preparations. Therefore, further studies using purified enzymes and advanced in vivo models are required to confirm the reproducibility and specificity of the observed inhibitory effects.

Currently, one pathological treatment strategy to inhibit tau hyperphosphorylation is GSK-3β inhibition, which is found to be hyperactivated in the brains of AD patients (Soni and Kaur, 2026).

In the present study, arbutin’s inhibitory activity against GSK-3β was measured with the Kinase-Glo luminescent assay, which quantitatively assesses ATP consumption as a measure of kinase inhibition. Although arbutin displayed lower potency compared to the broad-spectrum kinase inhibitor staurosporine as a positive control, the results obtained clearly indicate that arbutin possesses measurable inhibitory activity against GSK-3β. According to our findings, the potential of arbutin against GSK-3β was in parallel with the inhibitory activity of natural GSK-3β inhibitors such as flavonoids. It has been shown that flavopiridol and its derivatives inhibit GSK-3β, with IC_50_ values ranging between 0.059 and 1.184 μM, while arbutin’s inhibitory potential toward GSK-3β is higher than that of various phenolic compounds, with IC_50_ values ranging between 135 and 239.2 μM (Montanari et al., 2022).

Although Lineweaver–Burk plots remain widely used for preliminary characterization of enzyme inhibition mechanisms, reciprocal transformations may amplify experimental variability, particularly at low substrate concentrations. Therefore, the kinetic interpretations presented herein should be considered supportive rather than definitive, and further analyses using complementary kinetic approaches may provide additional mechanistic validation.

It is worth noting that the inhibitory potency of the compound revealed, together with its mechanistic kinetic picture and binding mode described in the present study, indicates its potential as a lead scaffold for further optimization efforts. Taken together, the current studies provide evidence for inhibitory activity of arbutin towards GSK-3β in vitro and thus justify further structural optimization and selectivity profiling. Kinetic studies showed a competitive pattern of inhibition with ATP and a K_i_ value of 0.03962 μM, which also suggests high affinity between the inhibitor and the active site. The noncompetitive nature of GSK-3β inhibition with respect to GS-2 is consistent with the inhibitor being bound at a site different from the substrate-binding site. This method of inhibition can be especially useful for targeting kinases that do not require threshold levels of intracellular substrate to operate with full catalytic activity. Given the regulation of GSK-3β and its roles in various signaling pathways, allosteric inhibition may offer advantages over ATP- or substrate-competitive inhibitors in terms of selectivity and off-target effects. These findings support the potential of arbutin as a modulator of GSK-3β activity through a substrate-independent mechanism.

The cytotoxicity data indicate that arbutin, when applied alone to SH-SY5Y cells, does not affect cell viability and can support cell viability and health. The absence of cytotoxic activity within the concentration range investigated supports the view that it can be a reliable compound for cell culture studies. Although the 24-h cytotoxicity effects of arbutin on SHSY-5Y cells were investigated, future studies necessitate longer exposure to β-arbutin to analyze the overall impact. Our findings align with the study in which α-arbutin extracted from Ericaceae species at a high experimental concentration of 500 μM showed negligible toxicity to SH-SY5Y cell lines (Ding et al., 2020).

Research on the docking of arbutin with GSK-3β indicates that a hydroxyl group from the D-glucose of arbutin coordinates with Mg^2+^ and interacts with ARG141. The hydroquinone moiety forms a hydrogen bond with VAL135. Previously, it was reported that ILE62, VAL70, ALA83, VAL110, LEU132, and LEU188 of GSK-3β form a hydrophobic pocket around adenine (Aoki et al., 2004). Similar residues were also observed here around arbutin, indicating that it is positioned in the hydrophobic pocket surrounding ADP, whereas ARG141 is among the residues for specific ATP/ADP recognition of GSK-3β (Aoki et al., 2004). In the present study, it is one of the key amino acids involved in the arbutin–GSK-3β interaction. A previous study demonstrated that the protonation state of GLU202 does not alter the AChE binding mode but influences the catalytic triad HIS447 activity (Wang et al., 2022). In the current study, this residue is also protonated (GLH202) and it engages in a hydrogen bond with arbutin, which is likely to affect AChE activity. Along with PHE338, this residue was also implicated in rivastigmine binding to AChE (Saleem et al., 2020).

In the present study, docking analysis showed binding of β-arbutin to the target enzymes with several noncovalent interactions, yet it predicts the interactions between ligand and protein and does not provide dynamic analysis. Key concerns about this approach can be eliminated through validation of the results with molecular dynamics, as well as cell culture/enzyme studies. Here, enzyme inhibition and cell culture studies show the overall effects of the chosen ligand, β-arbutin, compared to control compounds, partly supporting the results of the docking study.

## 5. Conclusion

Our findings demonstrate that arbutin exhibits inhibitory activity against cholinesterases and GSK-3β, suggesting its potential as a multitarget-directed natural compound for AD. However, since the present study is limited to in vitro and in silico analyses, further in vivo and clinical investigations are required to confirm its therapeutic efficacy and safety.

## Figures and Tables

**Table:** Inhibition of AChE, BuChE, and GSK-3β by arbutin.

	AChE IC_50_ (μM)	BuChE IC_50_ (μM)	GSK-3β IC_50_ (μM)
Arbutin	0.6 ± 0.4	4 ± 0.4*	1.23 ± 0.03**
Galantamine hydrobromide	0.69 ± 0.2	2.83 ± 0.3	-
Staurosporine	-	-	0.0363 ± 0.001

Data are presented as mean ± SD from three independent experiments. Statistical significance between arbutin and corresponding reference inhibitors was evaluated using Welch’s t-test assuming unequal variances.

* p < 0.05 and

** p < 0.001 were considered statistically significant.

## Data Availability

The data are available from the corresponding author upon suitable request.
